# Maternal death surveillance and response in Tanzania: comprehensiveness of narrative summaries and action points from maternal death reviews

**DOI:** 10.1186/s12913-020-06036-1

**Published:** 2021-01-11

**Authors:** Ali Said, Andrea B. Pembe, Siriel Massawe, Claudia Hanson, Mats Malqvist

**Affiliations:** 1 Department of Obstetrics and Gynaecology, Muhimbili University of Health and Allied Sciences, Dar es Salaam, Tanzania; 2 Department of Women’s and Children’s Health, Uppsala University, Uppsala, Sweden; 3 Department of Global Public Health, Karolinska Institutet, Stockholm, Sweden; 4 Department of Disease Control, London School of Hygiene and Tropical Medicine, London, United Kingdom

**Keywords:** Maternal mortality, Three phases of delays, Action plans, SMART, Maternal Death Surveillance and Response (MDSR), Death review, Narrative summary

## Abstract

**Background:**

Maternal deaths reviews are proposed as one strategy to address high maternal mortality in low and middle-income countries, including Tanzania. Review of maternal deaths relies on comprehensive documentation of medical records that can reveal the sequence of events leading to death. The World Health Organization’s and the Tanzanian Maternal Death and Surveillance (MDSR) system propose the use of narrative summaries during maternal death reviews for discussing the case to categorize causes of death, identify gaps in care and recommend action plans to prevent deaths. Suggested action plans are recommended to be Specific, Measurable, Attainable, Relevant and Time bound (SMART). To identify gaps in documenting information and developing recommendations, comprehensiveness of written narrative summaries and action plans were assessed.

**Methods:**

A total of 76 facility maternal deaths that occurred in two regions in Southern Tanzania in 2018 were included for analysis. Using a prepared checklist from Tanzania 2015 MDSR guideline, we assessed comprehensiveness by presence or absence of items in four domains, each with several attributes. These were socio-demographic characteristics, antenatal care, referral information and events that occurred after admission. Less than 75% completeness of attributes in all domains was considered poor while 95% and above were good/comprehensive. Action plans were assessed by application of SMART criteria and according to the place of planned implementation (community, facility or higher level of health system).

**Results:**

Almost half of narrative summaries (49%) scored poor, and only1% scored good/comprehensive. Summaries missed key information such as demographic characteristics, time between diagnosis of complication and commencing treatment (65%), investigation results (47%), summary of case evolution (51%) and referral information (47%). A total of 285 action points were analysed. Most action points, 242(85%), recommended strategies to be implemented at health facilities and were mostly about service delivery, 120(42%). Only 42% (32/76) of the action points were deemed to be SMART.

**Conclusions:**

Abstraction of information to prepare narrative summaries used in the MDSR system is inadequately done. Most recommendations were unspecific with a focus on improving quality of care in health facilities.

## Background

Worldwide, maternal mortality is still unacceptably high with about 295,000 maternal deaths in 2017[1]. Most of these deaths occur in low-resource countries both in and outside health facilities. Maternal death reviews have been done in many countries, including Tanzania, to reveal the causes and contributing factors to maternal deaths, with some success and challenges [2–7]. In 2015, Tanzania introduced the Maternal Death Surveillance and Response (MDSR) system in line with recommendations from the World Health Organization (WHO) [8, 9]. It is one way to address high maternal mortality, by developing context specific solutions to the identified problems and to guide national strategies towards improving quality of care. The MDSR system includes identifying, notifying and reviewing all maternal deaths to describe: (a) medical causes of deaths, (b) shortcomings/delays in the health system that contributed to the death, and (c) recommendations to address the identified delays.

**Fig. 1:**
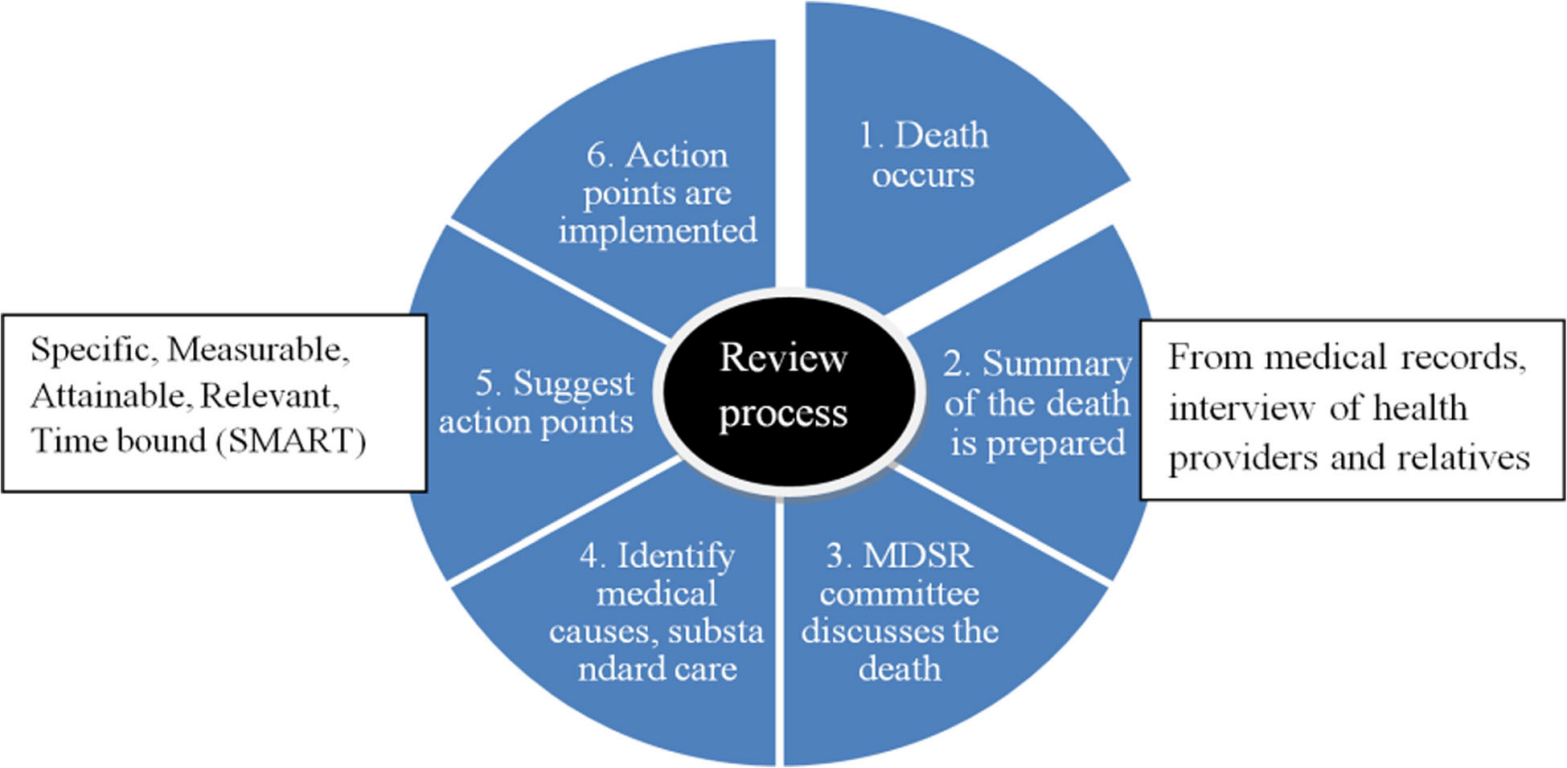
The process of maternal death review by MDSR committee

Facilities providing childbirth services in Tanzania are expected to have a multi-disciplinary MDSR committee to review all maternal deaths. The Tanzanian 2015 MDSR guideline includes instructions and illustrations on the collection of information from (i) medical records, (ii) interviews of health care providers and (iii) interviews of relatives who cared for the woman before death [8]. The information is used to prepare a narrative summary for discussion during MDSR meetings at health facilities, districts and sometimes regional levels of the health system. More information is sought in medical files (when available) during the meetings if what is written in the summary is not sufficient. A designated person in each facility is responsible for keeping the summaries confidential and they are kept as either hard or electronic copies. After the meeting, one or more recommended action points are suggested by the committee and filled into the Ministry of Health maternal death reporting form. The action points are meant to stir up action at both local and national levels to prevent future maternal deaths [10]. The Tanzanian 2015 MDSR guideline recommends that action points from maternal death reviews should have clearly defined and measurable activities so that implementation can be tracked and assessed. That means they should be *Specific, Measurable, Attainable, Relevant and with specific allocated Time* (SMART), as well as appoint a responsible person for the implementation. (See Fig. 1). The action points are supposed to be shared with the district health office and quality improvement committees for further follow up. Having a system which allows for the following up on the quality and implementation of recommended action points can be effective in making sure MDSR is implementable. A study from Nigeria which reported use of a scorecard to track MDSR implementation identified facilities making recommendations without clearly defined action points [10]. This may have created problems during implementation and follow up of the action points. The Tanzanian MDSR guideline of 2015 does not provide a framework for follow up on the implementation of action points but recommends the development of SMART plans.

Comprehensive documentation of history, physical examination, investigation results and treatment in medical practice is important in assisting practitioners and other medical staff to manage and follow up patients, as well as to use the information in research and audits/reviews to improve practice and patients’ safety [11–13]. During death reviews such as those in the MDSR system, the quality of documents used may directly impact the recommendations from the audit, especially when it is done from abstracted information. Heath care provider`s failure to follow guidelines when prioritizing care over documentation may lead to inadequate abstracted information [14]. Studies from the United States and Iran have shown health care providers` failure to follow recommendations has led to the poor gathering and storing of medical records [12, 15–17]. A report on MDSR implementation as part of a Maternal and Child Survival program in Northern Tanzania revealed that most facilities` medical records were not sufficient to attribute the exact cause of death and identify concretely the area in which substandard care had been given [18].

Health facilities in Tanzania face challenges in medical files documentation and record keeping. The narrative summaries used in reviews are expected to be more comprehensive since the preparation process involves going through multiple sources. It is therefore important to investigate the quality of the narrative summaries in order to explore opportunities for improvements.

We sought to investigate the availability and comprehensiveness of the summaries in health facilities, and assess how well action plans aligned with the SMART criteria. Results will provide recommendations for improvement of record keeping and gathering of information in the narrative summaries of maternal deaths.

## METHODS

### Study design

This was a retrospective desk review of maternal deaths documents (narrative summaries and action plans). To do this we visited all facilities that reported deaths and reviewed their narrative summaries and action plans. We included 122 maternal deaths that had occurred between January 1st to December 31st, 2018 in the Mtwara and Lindi regions, of Southern Tanzania.

### Study setting

The total population of the Lindi and Mtwara regions is about two million people [19]. There are two regional referral hospitals, eight district hospitals, four private/mission hospitals, 40 health centres and 399 dispensaries. Even though the regions have a total of 15 districts, there are only eight district hospitals. Other districts are served by a health centre or district designated hospital as the main referral facility in the district. In 2013, the Maternal Mortality Ratio (MMR) was 456 in Lindi and 579 per 100,000 live births in Mtwara according to census data [20]. Facility delivery was 80.8% and 81.3% respectively, caesarean section rate 6.0% and 10.3% respectively and family planning use was at least 50% in both regions according to the Tanzania Demographic Health Survey of 2015[21]. The two regions, similar as all other regions in Tanzania, implemented the MDSR system as recommended in the 2015 guideline.

### Outcomes

We reviewed the narrative summaries of maternal deaths by following a defined set of criteria. We defined the *Comprehensiveness* of narrative summaries as those that contained 95% or more of the recommended information. A checklist informed by recommendation in the Tanzanian 2015 MDSR guideline was prepared for data collection [8].The information in the checklist was divided into four domains each with several attributes (Panel 1). The domains were (1) Demographic characteristics and Antenatal care information (12 attributes), (2) Delivery/abortion information for those who delivered/aborted before admission (six attributes) (3) Referral information (four attributes) (4) Information on events after admission (20 attributes).

**Panel 1: Domains and attributes checked to assess the comprehensiveness of the narrative summaries**.

**1. Demographic characteristics and Antenatal care information**.

- Date of review, Maternal death review number, Patient code, Age, Marital status, Gravidity, Parity, Live children, Mode of delivery of previous pregnancy, Date of last caesarean section, Number of antenatal care visits in this pregnancy, Risk factors detected during this pregnancy.

**2. Delivery/abortion information for those who delivered/aborted before admission**.

- Date of delivery/abortion, Duration of amenorrhea, Status of baby at delivery (dead/alive/abortion), Place of birth/abortion(home/facility), Assisted by who, Information on complications that occurred after delivery..

**3. Referral information**.

- Type of referring facility, Reason for referral, History of the case, How does a woman’s position in the community affect her referral..

**4. Information on events after admission**.

- Date of admission, Main reason for admission, Summary of history, physical examination and investigations, Initial diagnosis at admission, Summary of case evolution, Sequence of events of abortion/delivery, Indication of surgery, Diagnosis made at complications, How does a woman’s position in the community affect process after admission, Treatments given, Time between diagnosis of complication and treatment, Complementary Investigation results present, Summary of case evolution (monitoring vital signs, input output, bleeding), Date of Death, Time between complications and death, Cause of death, Pregnancy outcome, Other information (from community or other centres)..

A SMART action point means that a recommended action point is *Specific, Measurable, Attainable, Relevant and Time-bound*. An action was considered *Specific* if it clearly mentioned what is to be done, how it will be done, who will do it and describes the expected results of the action point. An action point was considered *Measurable* if it could be evaluated against standards. *Attainability* meant that the action could be implemented considering the resources and available skills and capacity. A *Relevant* action was considered as an action that was actually needed considering the case and the dysfunction identified. An action was considered *Time-bound* when it had a specific time for starting, ending or both.

### Data sources and measurements

In March and April 2019, a team of researchers led by the first author (AS) visited all the health facilities that reported a maternal death in the year 2018.Upon visiting the facilities, the researchers requested documents of the reported maternal deaths from the facility in-charge. The team also requested the narrative summaries and action plans of all 122 reported maternal deaths that were reviewed in line with the MDSR. The regional data revealed that 23 maternal deaths occurred within the two regional hospitals, 54 deaths across all eight district hospitals, 31 deaths across three mission hospitals, 12 deaths across nine health centres and 2 deaths within two dispensaries. In most facilities the documents were kept separately from hospital medical records by the facility matron or the District Reproductive and Child Health Coordinator (DRCHCo). The researchers were directed to their offices to retrieve them. The researchers were able to retrieve 76 summaries which had 285 action points.

The first author (AS) reviewed the narrative summaries by familiarisation and checking for presence of attributes on the four different domains (Panel 1). Presence or absence of information/attributes in each domain was scored and coded as present (1), not present (2) or not applicable (3) depending on the case. The researcher read each summary repeatedly to make sure all information was available or not, even if it was not explicitly mentioned. For example, the duration of amenorrhea was considered to be present if the last normal menstrual period or gestation age (in week/months) was mentioned. Also, marital status was considered to be present if it was mentioned that the deceased was brought to the facility by her husband.

After familiarisation with the action plans the first author extracted (i) the target of each action point (community, facility or higher level), (ii) specific issues it addressed in the community or facility. For community action points, the researcher indicated whether the action point was for decision making at family level, danger signs recognition, health seeking behaviour or traditional practices. Action points in the health facility were assessed to determine whether they addressed service delivery, human resource, equipment and supplies, referral system, accountability or facility infrastructure. The action points were then assessed for appropriateness by checking whether they met the SMART criteria.

### Quantitative variables

Quantitative data collected was entered and cleaned in IBM SPSS Statistics for Windows version 23 (IBM corp., Armonk, N.Y., USA) for analysis. The *Comprehensiveness* of each narrative summary was determined by calculating the individual proportion of amount of information depending on each case. For each summary, the total amount of present information was calculated. This was then divided by the total amount of expected information for each summary to get the proportion of present information. The proportional score of each summary was ultimately categorised as *poor, average, or good/comprehensive* if it had 0–74%, 75–94% or 95% and above of the required information respectively. Four attributes were removed from the final analysis since they were too ambiguous and most summaries had collected no information on them. The cut off points were based on a previous study done by Mohseni et al. in Iran [15]. These cut offs were used for analysis and description purposes only and therefore are not recommended as standard cut off levels. Action points were considered to be SMART if all the criteria were met.

### Statistical methods

Descriptive analysis was done for all the variables and data presented in the figures and tables.

## Results

Narrative summaries were available for 76(62%) of maternal deaths from both regions. The missing summaries were lost due to poor record keeping, as summaries were often removed from facilities to be used in district and regional meetings. Some were probably lost because the facilities had reshuffled offices and providers who kept the summaries lost them when they changed offices. Furthermore, some deaths that were available in regional data could not be found in facility records where they were reported to have occurred.

### Assessment of the comprehensiveness of the narrative summaries

Each narrative summary is recommended to include demographic, antenatal care information, delivery information (if delivered before admission), referral information (for referred cases), and information on events after admission until death.

Age and gravidity were the most common information 69(91%) present in the summaries, while only 7(9%) of the summaries had a maternal death review number indicated. Only 8(13%) summaries indicated the mode of delivery of previous pregnancy and only one had a date of previous caesarean section. (Table 1)

**Table 1:** Assessment of the presence of demographic characteristics, antenatal care, delivery/abortion and referral information

Variable	Frequency	Percent
**Demographic and antenatal care information (*N* = 76)**		
Date of review	18	23.7
^c^Maternal death review number	7	9.2
Patient code	8	10.5
Age	69	90.8
Marital status	8	10.5
Gravidity	69	90.8
Parity	68	89.5
Live children	54	71.1
Mode of delivery of previous pregnancy^a^	8	12.7
Date of last caesarean section^b^	1	25.0
Number of antenatal care visits in this pregnancy	54	71.1
Risk factors detected during this pregnancy	46	60.5
**Delivery/abortion information for those who delivered before last admission (*N* = 19)**		
Date of delivery/abortion	18	94.7
Duration of amenorrhoea	8	42.1
Status of baby at delivery (dead/alive/abortion)	11	57.9
Place of birth/abortion(home/facility)	18	94.7
Assisted by who	11	57.9
Information on complications	16	84.2
**Referring information for referral cases (*N* = 32)**		
Type of referring facility	28	87.5
Reason for referral	26	81.3
History of case	17	53.1
^c^How does a woman’s position in the community affect her referral	0	0

^a^Only for eligible cases (multigravida) *N*=63

^b^Only for eligible cases (those reported C/S for previous delivery) *N*=4

^c^Removed from analysis of Comprehensiveness

The table also indicates that for those who delivered before the last time they were admitted 18(95%) of their summaries had information on date and place of delivery/abortion while 8(42%) had information on the duration of amenorrhea.

Most summaries 28(88%) indicated the type of referring facility, while none of them indicated “how the woman`s position in the community affects her referral” as recommended in the guideline.

Date of admission, main reason for admission, summary of case evolution, sequence of delivery/abortion events, surgery indication and date of death were present in more than 94% of summaries (Table 2). Information on how the woman`s position affects the process after admission was not present in any of the summaries. Overall, 64(84%) of summaries were scored to be *poor* and only 12(16%) were *average* and none were *good/comprehensive*. Some variables were considered not important since they were not present in most summaries and had ambiguous descriptions. When these four variables were removed (Tables 1 and 2) the final summaries score changed to 48.7% poor, 50% average and 1.3% comprehensive.

**Table 2:** Assessment of the presence of information on events after admission (*N* = 76)

Variable	Frequency	Percent
Date of admission	73	96.1
Main reason for admission	75	98.7
Summary of history, physical examination and investigations	70	92.1
Initial diagnosis at admission	67	88.2
Summary of case evolution	72	94.7
Sequence of events of abortion/delivery occurred^a^	62	95.4%
Indication of surgery written^a^	44	95.7
Is there diagnosis made at complications	61	80.3
Treatments given	66	86.8
Time between diagnosis of complication and treatment	49	64.5
Complementary Investigation results present^a^	36	47.4
Summary of case evolution (monitoring vital signs, input output, bleeding)	39	51.3
Date of death	73	96.1
Time between complications and death	62	81.6
Cause of death	67	88.2
Pregnancy outcome	67	84.2
^b^Other information (from community or other centres)	12	15.8
^b^How does woman’s position in community affect process after admission	0	0

^a^Only for eligible cases

^b^Removed from analysis of comprehensiveness

### Assessment of the recommended action points after maternal death reviews

**Fig. 2:**
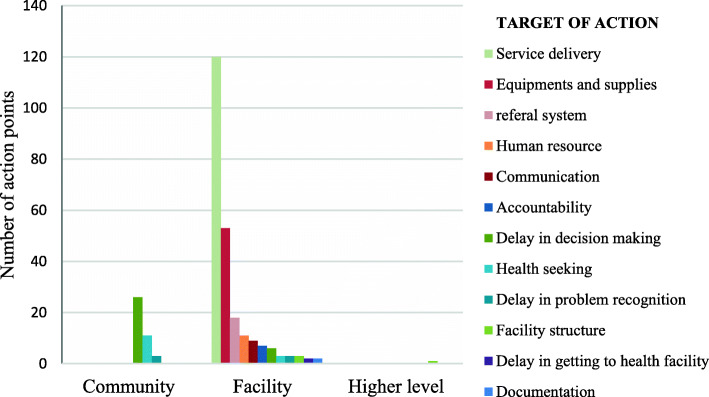
Place of implementation and issues addressed by the action points

A total of 285 action points were included in the analysis. Data on the implementation of each action point was not available, so it is not reported in this study. This is because facilities could not provide written evidence of implementation of each action point. Out of the reviewed action plans, 242(85%) action points targeted the facility level, 42(15%) the community level and 0.4% higher levels of health systems. Almost half 120(42%) of the action points directed to the facility were for service delivery, such as knowledge and skills, while at the community level most action points were for delays in decision making (Fig. 2).

### Recommended action points assessment

Two summaries did not have documented action points. A total of 285 recommended action points were included in the analysis.

Table 3 shows that approximately 42% of the action points were SMART, most of them were time bound (93%) and (71%) were deemed relevant.

**Table 3:** Assessment of the action points using SMART criteria (n = 285)

Variable	Frequency	Percent
Specific	131	46.0
Measurable	146	51.2
Achievable	184	64.6
Relevant	201	70.5
Time	265	93.0
SMART action point	119	41.8

## Discussions

### Main findings

Our study reveals that only 62% of narrative summaries for maternal deaths were available and only 1% had most (more than 95%) of the recommended information in accordance to the 2015 MDSR guideline. Missing key information included information on events that occurred in the community before reaching facility (16%), time between diagnosis of complication and commencing treatment (65%), investigation results (47%) and summary of case evolution after complications (vitals, input, output, treatments given) (51%). Furthermore, just over half of referral deaths had summary of the medical history, physical examination and treatment of case before referral (53%). Demographic characteristics such as death review number, patient code, marital status, duration of amenorrhea and mode of delivery of previous pregnancy were missing in most summaries. Most action points (85%) were directed towards health facilities and were mostly targeting service delivery issues such as knowledge and skills that caused human errors in management. Only 42% of the action points were deemed to be SMART, most of the action points (93%) had a timeline of implementation while less than half (46%) were found to be specific.

### Availability of narrative summaries

Only two-thirds of the expected narrative summaries were available in the visited facilities, even though all deaths were reported to have been reviewed. The summaries were most likely lost due to poor record keeping and the removal of documents from the facility for district and regional meetings. This could have been mitigated by using electronic record keeping or having multiple hard copies of the summaries in the facilities.

### Comprehensiveness of the narrative summaries and action plans

Our study indicates that the MDSR systems are constrained by poorly prepared narrative summaries which are lacking important information. One of the reasons for poorly prepared summaries could be the quality of information recorded in medical records, since these are the main source of data for summaries. This has also been reported in studies done in high, middle and low-income countries. Studies in the US, Wales, and the UK have shown that medical records have poorly documented general symptoms, gynaecological history, treatment side effects, smoking history and drug allergies [16, 22, 23]. Furthermore, the confidential enquiries to maternal deaths in the UK have also been reported to face challenges in obtaining medical record and short reports, especially from general practitioners [24]. The situation is worse in African and other low and middle-income countries which are still struggling to establish electronic medical records that are better than hand written records [15, 25, 26]. Health providers should also understand documentation of medical records is as important as providing health services [14]. Poorly documented medical records have a direct negative impact on the comprehensiveness of summaries abstracted from such documentation. Luck et al. in a study on quality of abstracted information in general internal medicine patients, cautioned against measuring quality using abstracted information due to deficiencies in preparing summaries [14]. They reported that records’ abstraction resulted in providing only 54% of the standard information required. Furthermore, a study on identification of causes and three delays in the MDSR system in Tanzania revealed that when using narrative summaries, the providers failed to identify most of the gaps in care that contributed to that death [27]. This also impacted the action plans from such reviews.

Another reason for poorly documented narrative summaries in our study could be the fact that in some health facilities the person who was involved in the management of the deceased is tasked with writing the summary. This could lead to attempts of hiding some of the information within the summary for fear of blame. A study in Malawi revealed that fear of blame was one of the main barriers to conducting maternal deaths reviews in health facilities [28]. This problem could have been mitigated by providing an example of a comprehensive narrative summary in the guideline, or using a checklist box for the provider to fill in the required information [8]. Facilities should also create a supportive environment by addressing fear of blame and provide clear user-friendly guidelines for doctors and other health providers, so they can take an active part in summary writing. This will help the summaries to be more comprehensive and provide opportunity for learning and attitude change among doctors and other providers.

The guideline also had items that were ambiguous and not well understood affecting further the quality of the summaries. The items “How does a woman`s position in the community affect her referral or process after admission” held unclear meanings, and providers writing the summaries could have had difficulties understanding what it meant. This explains why the researchers could not find any information in the summaries addressing these items. The guide should be revised to make sure the items are relevant and measurable for providers and researcher to understand them.

Documented recommendations or action plans in the MDSR systems were mostly directed to health facilities (third delay), targeting directly health care provision such as knowledge and skills of health care providers. This seems to be reasonable as also other studies from Malawi, Tanzania, Kenya and Nigeria indicated that most maternal deaths occur due to substandard care in health facilities [2, 3, 29–31], while facility delivery in these countries stands at 91%, 63%, 61% and 39% respectively [21, 32–34].

For the action plans to be effective in preventing and reducing maternal death they need to be implementable and easy to follow up. Most action points in the MDSR system were found to be non-specific (54%) as they were not clear about what was going to be done and only 42% were found to be SMART. This may limit the impact of the MDSR strategy on quality of care in Tanzania. A few studies have assessed the recommended action plans in the MDSR system such as in Nigeria and in Northern Tanzania [10, 18]. During maternal deaths review, health care providers should develop recommendations with implementation plans in mind.

The implementation of action plans is the most important step, signifying the response part of the MDSR system. This study was not able to assess the implementation of these actions because most facilities did not have a good and systematic way of tracking the implementation of each action point. The system should be reviewed to make sure the action plans are implemented.

### Limitations of the study

The main limitation of this study is the fact that the summaries were assessed by one person (AS). Bias was however minimized by using a prepared checklist constructed from the MDSR guideline’s recommendations. The cut-off points used in the analysis of information were informed by a study from Iran [15] but we acknowledge that they are arbitrary. This is because the study in Iran was not based on summaries of maternal deaths but rather the comprehensiveness of information within medical file records. In our study we used them for description purposes only and do not indicate standards in amount of medical information documented. A good narrative summary should include all, or almost all essential points, and a lower cut-off would have given a wrong impression. The comprehensiveness of the summary is important inorder to make a diagnosis and create a good action plan. This is also subjective and depends a lot on the expertise of the death reviewer. One of the objective ways to assess the summaries was to score them for descriptive purposes. The generalisability of the study in settings where the MDSR system does not use narrative summaries is also a limitation. Even in these settings, the study informs the importance of measuring quality of care using comprehensive medical information. It also shows the effects of incomplete documentation of medical information as it affects the quality of abstracted information.

## Conclusions

Abstraction of information to prepare narrative summaries used in the MDSR system is inadequately done. This can negatively impact the relevance and quality of recommendations developed using the summaries. Most recommendations focus on improving quality of care in health facilities but are not specific on the issues to be addressed.

## Recommendations

To improve documentation of narrative summaries and recommended action plans, providers should use a checklist with spaces in which required information could be filled in. A scheduled follow up of action point implementation is needed to ensure reviews work as intended.

Further qualitative studies among health care providers are needed to explore challenges and solutions to help them write better summaries. There is also a need to study ways to improve action plan formulation and to what extent they are implemented. Furthermore, research is needed to explore how to improve the overall MDSR system in Tanzania.

## Data Availability

Datasets used and/or analysed during the current study are available from the first author on request.

## Abbreviations

C/S: Caesarean Section
MDSR: Maternal Death Surveillance and Response
MMR: Maternal Mortality Ratio
SMART: Specific, Measurable, Attainable, Relevant and Time-bound
WHO: World Health Organization
